# Paradoxical Myocardial Infarction After Thrombolytic Therapy for Acute Ischemic Stroke

**DOI:** 10.7759/cureus.16659

**Published:** 2021-07-27

**Authors:** Billal Mohmand, Abeeha Naqvi, Avneet Singh

**Affiliations:** 1 Internal Medicine, Upstate University Hospital, Syracuse, USA; 2 Internal Medicine, Syracuse Veterans Affairs Medical Center, Syracuse, USA; 3 Cardiology, Upstate University Hospital, Syracuse, USA

**Keywords:** acute st-elevation myocardial infarction, myocardial infarction, : acute coronary syndrome, tissue plasminogen activator (tpa), cerebro-vascular accident (stroke), : ischemic stroke, st-elevation myocardial infarction (stemi), systemic thrombolysis, coronary artery angiography, complication of treatment

## Abstract

Early reperfusion therapy with tissue plasminogen activator (tPA) for acute ischemic stroke has mortality benefits despite the risks. Myocardial infarction (MI) after the use of thrombolytic therapy is a rare complication. We report a 67-year-old woman with acute stroke who received tPA for acute ischemic stroke and subsequently developed ST elevation MI (STEMI), highlighting a rare and serious complication.

## Introduction

Early reperfusion therapy with tissue plasminogen activator (tPA) for acute ischemic stroke has long-term mortality benefits despite known risk factors [1]. Additionally, it is a viable option for ST elevation myocardial infarction (STEMI) when there is limited access to percutaneous coronary intervention (PCI). 

Myocardial infarction (MI) after tPA use is a rare complication with unknown incidence and rarely reported [2]. Our case represents the appropriate use of tPA for acute ischemic stroke per the inclusion criteria which is well documented in the literature, however, with an unexpected development of acute MI immediately following tPA administration. Despite this rare adverse effect, our case was unique from other reports as our patient had a positive outcome, while similar cases had fatal outcomes [3]. 

## Case presentation

**Figure 1:**
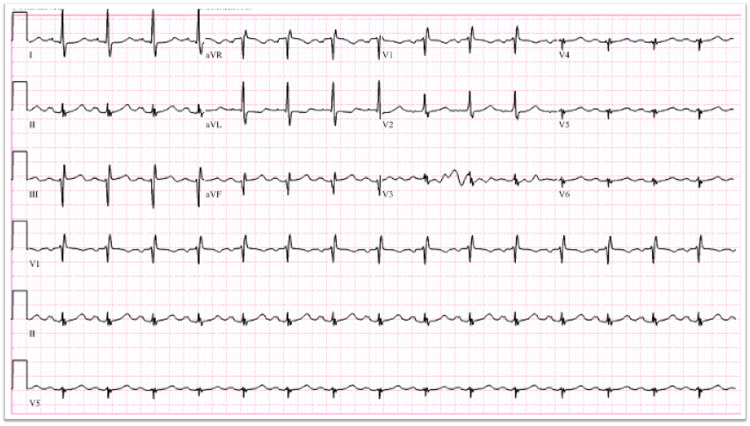
Electrocardiogram with inferior lead ST elevations and reciprocal depressions after tPA administration. tPA, tissue plasminogen activator

A 67-year-old female who had a medical history significant for coronary artery disease with PCI three years prior [drug eluting stent (DES) to the left anterior descending artery], cerebrovascular accident six months prior, hyperlipidemia (HLD) with statin intolerance, and type II diabetes mellitus presented to an outside hospital with sudden onset left-sided hemiparesis and dysphasia. The patient was diagnosed with an acute stroke with a National Institutes of Health Stroke Scale score of three and received tPA with an improvement of her symptoms. Approximately one hour later the patient developed acute left-sided chest pressure, nausea, and emesis. Electrocardiogram (ECG) indicated new ST elevations in inferior leads with reciprocal ST depressions (Figure 1). Troponin I was found to be elevated to 0.13 ng/mL (normal: <0.04 ng/mL).

The patient received aspirin 325 mg and was started on heparin infusion for the acute coronary syndrome. The patient was transferred for presumed ST-elevation myocardial infarction (STEMI) to our institution. On arrival, she was noted to have some improvement in her chest pain. Troponin T on arrival was found to be elevated to 0.06 ng/mL (normal: <0.01 ng/mL) and subsequent monitoring found a peak level of 3.00 ng/mL (normal: <0.01 ng/mL) at 18 h since the event. Neurology recommended no anticoagulation or anti-platelet agents during the initial 24 h after tPA administration. Heparin was discontinued and cardiac catheterization was deferred for the first 24 h. 

**Figure 2:**
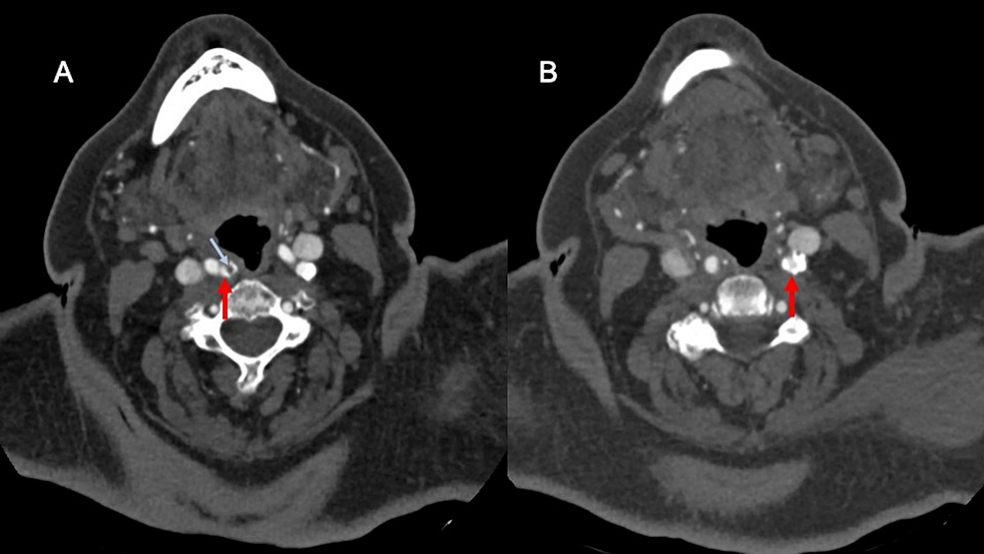
Axial contrast-enhanced CT images of the neck (A, B) demonstrate moderate stenosis of the proximal ICAs, as per NASCET criteria (red arrows in A and B). Additionally, there is a 5.6 mm ulcerated atheromatous plaque in the proximal right ICA (blue arrow in A). ICA, internal carotid artery

**Figure 3:**
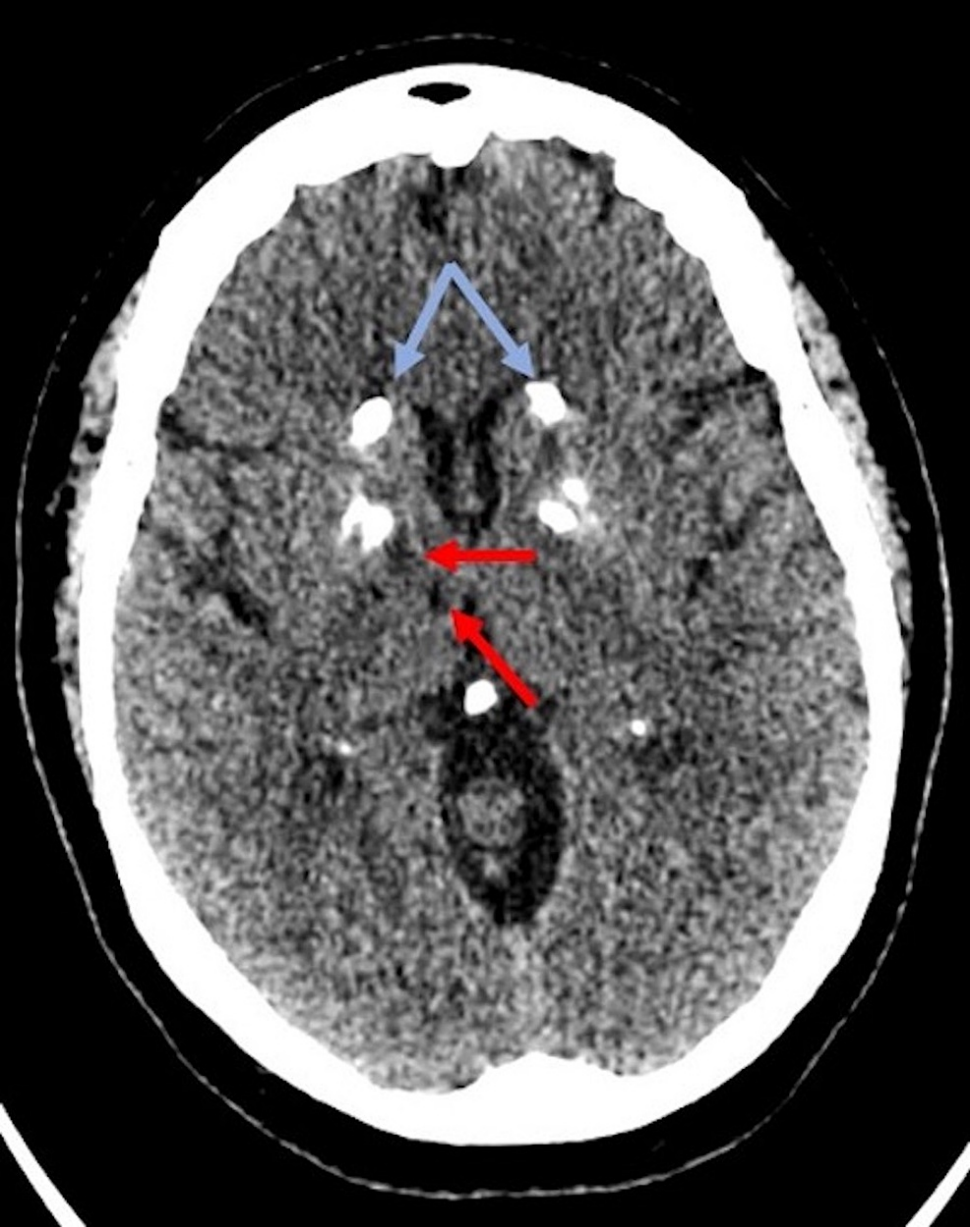
Non-contrast axial CT image of the head demonstrating subtle foci of hypo-attenuation in the region of the anterior right thalamus (red arrows). Bilateral basal ganglia calcifications are noted (blue arrows).

**Figure 4:**
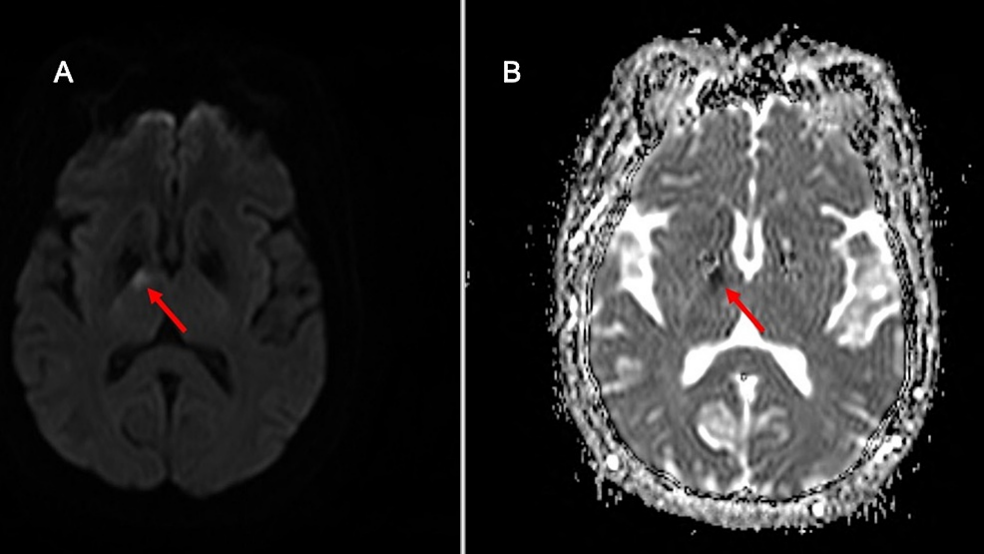
Axial (A) DWI with corresponding (B) ADC map of the brain confirm the area of ischemia in the anterior right thalamus as depicted by the hyper-intense and concurrent hypo-intense signal on the DWI and ADC images, respectively (red arrows). DWI, diffusion weighted image; ADC, apparent diffusion coefficient

The patient underwent CT angiography neck which revealed moderate bilateral internal carotid artery (ICA) stenosis with a 5.6 mm predominantly noncalcified ulcerative plaque in the proximal right ICA (Figure 2). Non-contrast CT head demonstrated subtle foci of hypo-attenuation in the anterior right thalamus (Figure 3). MRI of the brain indicated an acute ischemic infarct of the right anterior thalamus (Figure 4).

Due to concern for further unstable plaques, atorvastatin 80 mg while awaiting cardiac catheterization. Liver functions were subsequently elevated from normal levels to an Aspartate amino-transferase level of 167 U/L (normal: <32 U/L) and Alanine amino-transferase level of 56 U/L (normal: <32 U/L), concerning hepatotoxicity and no further statins were given. Additionally, echocardiography was completed during the waiting period which indicated normal systolic function with 61% ejection fraction with no inter-atrial shunt.

**Figure 5:**
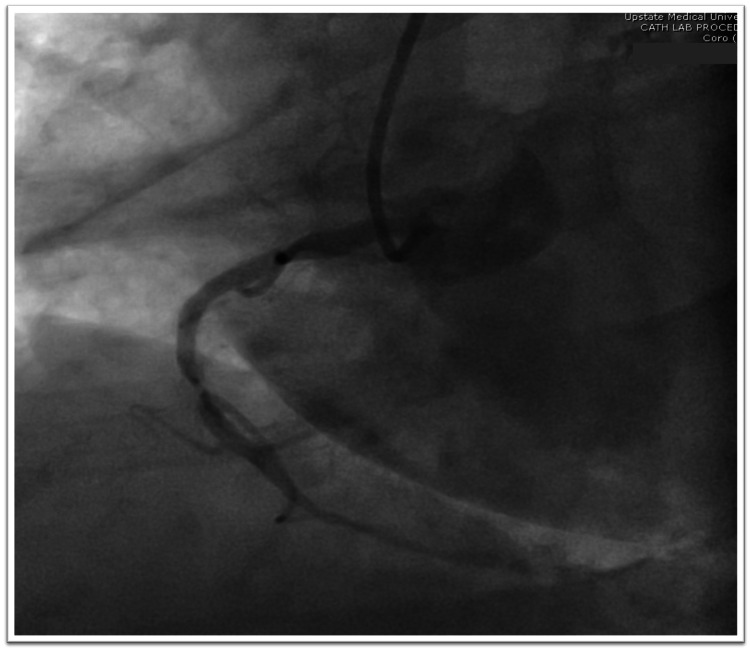
Coronary angiography revealing 90% stenosis in the RCA. RCA, right coronary artery

**Figure 6:**
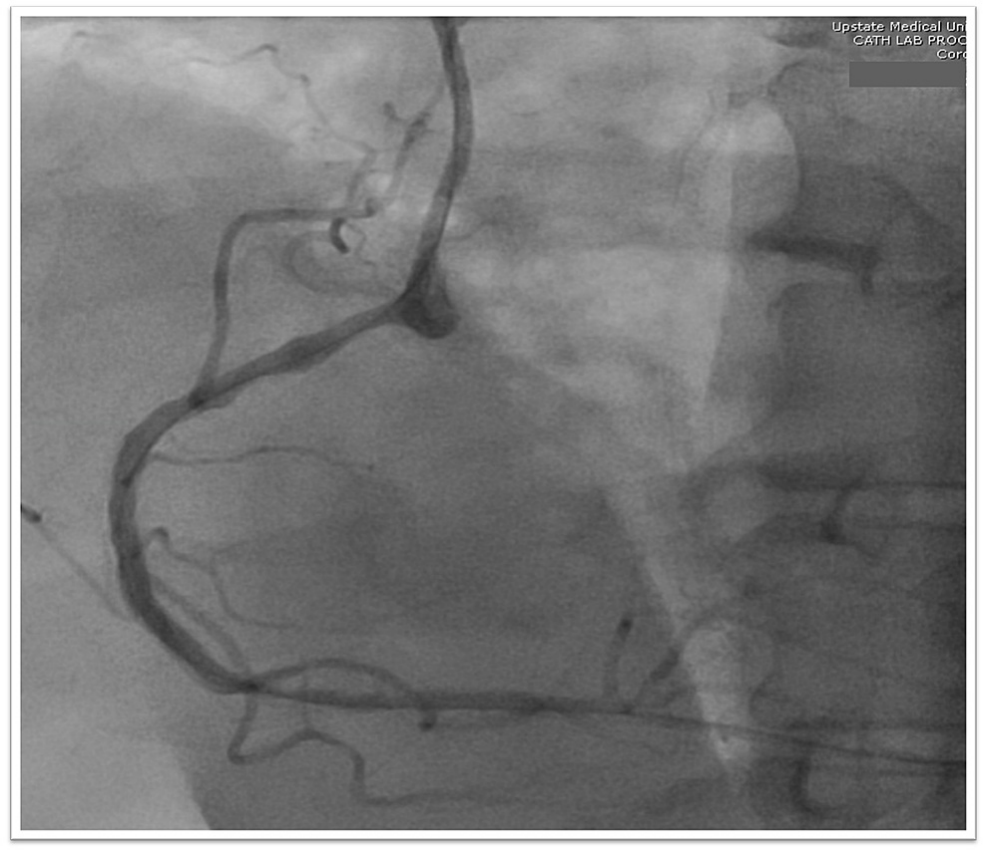
Percutaneous coronary intervention with DES placement in the RCA. DES, drug eluting stent; RCA, right coronary artery

After completion of the initial 24 hour waiting period from tPA administration, and an unremarkable repeat CT head, the patient underwent cardiac catheterization which found a 90% stenosis in the right coronary artery (Figure 5). The patient was treated with a DES (Figure 6) for the significant stenosis seen on cardiac catheterization.

The patient was started on dual anti-platelet therapy, aspirin and clopidogrel, and continued on guideline-based therapy with metoprolol succinate and lisinopril. Due to the statin intolerance the patient was discharged with ezetimibe with outpatient evaluation for proprotein convertase subtilisin/kexin type 9 (PCSK9) inhibitors. The patient ultimately qualified for PCSK9 inhibitor for her uncontrolled HLD and tolerated the medication well with significantly improved lipid management. 

## Discussion

Our case highlighted a clear temporal relationship between tPA administration and the onset of STEMI. This unusual phenomenon was unique as the event was clearly delineated under direct observation. The patient was observed to have a normal ECG and troponin value during admission and initial evaluation for stroke. The rapid onset with ECG changes and troponin elevations support a STEMI event. The acute onset of MI within 2 h of thrombolytic therapy has been rarely documented in a few published cases [3-4]. 

A particularly complex aspect was the management decisions for the cardiac event after tPA administration where therapeutic guidelines recommend against anti-thrombotic treatment in the first 24 h after administration due to increased bleeding risks [5]. The decision was ultimately made in our case to defer cardiac catheterization as the patient was hemodynamically stable, reassuring echocardiogram findings and a Killip Class I score with low risk (2%-3%) of 30-day mortality [6]. However, if the patient were to have developed severe cardiogenic shock this may have led to a different prioritization of management.

Multiple pathogenic mechanisms for such an event have been suggested; we suspect our case to be related to a local thrombosis from an unstable plaque atheroma in a stenotic coronary artery leading to STEMI. Coronary angiography demonstrated severe stenosis of 90% in the RCA consistent with the ECG findings during the onset of chest pain. Similar reports have observed significant coronary stenosis presence, raising suspicion of a role in the mechanism of such incidents. The presence of significant unstable plaque atheroma was evidenced by imaging revealing noncalcified ulcerative plaques in the proximal right ICA. The lack of plaque stabilization effects from statins likely allowed the presence of further unstable plaques, particularly within the coronary arteries. Without the plaque stabilization and anti-inflammatory effects statins, our patient was vulnerable to plaque rupture, likely precipitated by tPA administration. 

Thrombolytic agents can lead to fragmentation and mobilization of pre-existing thrombi. Severe embolic complications have been observed in 1.5% of patients with thrombolytic use after acute myocardial ischemia, with many being fatal. Concern has been raised with the presence of pre-existing blood clots in the vascular system, particularly the left atrium, ventricular aneurysms, or aortic aneurysms, and whether thrombolytic therapy should be contraindicated [7]. In our particular case, there was no indication of the presence of pre-existing vascular blood clots on echocardiography or CT imaging, however, due to the cardiac catheterization being deferred due to the high risk, it cannot be definitively determined if there was the presence of emboli in the coronary arteries at the time of the event.

## Conclusions

Developing acute MI immediately following tPA administration is a serious complication and not well documented. Unstable atherosclerotic plaques may predispose patients to the development of tPA-induced MI. Interestingly, the reverse phenomena with tPA used for MI subsequently causing ischemic stroke have been readily documented. We suggest practitioners remain cognizant of this rarely documented yet potentially fatal adverse effect of tPA. 

## References

[1] National Institute of Neurological Disorders and Stroke rt-PA Stroke Study Group (1995). Tissue plasminogen activator for acute ischemic stroke. N Engl J Med.

[2] Manea MM, Dragoş D, Stoica E, Bucşa A, Marinică I, Tuţă S (2018). Early ST-segment elevation acute myocardial infarction after thrombolytic therapy for acute ischemic stroke: a case report. Medicine (Baltimore).

[3] Meissner W, Lempert T, Saeuberlich-Knigge S, Bocksch W, Pape UF (2006). Fatal embolic myocardial infarction after systemic thrombolysis for stroke. Cerebrovasc Dis.

[4] Sweta A, Sejal S, Prakash S, Vinay C, Shirish H (2010). Acute myocardial infarction following intravenous tissue plasminogen activator for acute ischemic stroke: an unknown danger. Ann Indian Acad Neurol.

[5] Powers WJ, Rabinstein AA, Ackerson T (2018). 2018 Guidelines for the Early Management of Patients With Acute Ischemic Stroke: A Guideline for Healthcare Professionals From the American Heart Association/American Stroke Association. Stroke.

[6] Killip T, Kimball JT (1967). Treatment of myocardial infarction in a coronary care unit. A two year experience with 250 patients. Am J Cardiol.

[7] Stafford PJ, Strachan CJ, Vincent R, Chamberlain DA (1989). Multiple microemboli after disintegration of clot during thrombolysis for acute myocardial infarction. BMJ.

